# Risk of thrombotic events after respiratory infection requiring hospitalization

**DOI:** 10.1038/s41598-021-83466-9

**Published:** 2021-02-18

**Authors:** Nathaniel R. Smilowitz, Varun Subashchandran, Jonathan Newman, Michael E. Barfield, Thomas S. Maldonado, Shari B. Brosnahan, Eugene Yuriditsky, James M. Horowitz, Binita Shah, Harmony R. Reynolds, Judith S. Hochman, Jeffrey S. Berger

**Affiliations:** 1 Leon H. Charney Division of Cardiology, Department of Medicine, Center for the Prevention of Cardiovascular Disease, New York University School of Medicine, 530 First Avenue, Skirball 9R, New York, NY 10016 USA; 2 Department of Medicine, VA New York Harbor Healthcare System, New York, NY USA; 3 Department of Surgery, New York University School of Medicine, New York, NY USA; 4 NYU Division of Pulmonary, Critical Care, and Sleep Medicine, Department of Medicine, New York University School of Medicine, New York, NY USA

**Keywords:** Myocardial infarction, Cerebrovascular disorders, Thromboembolism, Thrombosis, Infection

## Abstract

Thrombosis is a major concern in respiratory infections. Our aim was to investigate the magnitude and duration of risk for arterial and venous thrombosis following discharge after respiratory infection. Patients with respiratory infections were identified using the United States Nationwide Readmission Database from 2012 to 2014. Patients admitted with asthma or cellulitis served as comparators. Readmissions for acute myocardial infarction (MI) and venous thromboembolism (VTE) were evaluated at 30 to 180 days. The likelihood of a first thrombotic event after discharge was compared with a 30-day period prior to hospitalization. Among 5,271,068 patients discharged after a respiratory infection, 0.56% and 0.78% were readmitted within 30-days with MI and VTE, respectively. Relative to asthma and cellulitis, respiratory infection was associated with a greater age and sex-adjusted hazard of 30-day readmission for MI (adjusted HR [aHR] 1.48 [95% CI 1.42–1.54] vs. asthma; aHR 1.36 [95% CI 1.31–1.41] vs. cellulitis) and VTE (aHR 1.28 [95% CI 1.24–1.33] vs. asthma; aHR 1.26, [95% CI 1.22–1.30] vs. cellulitis). Risks of MI and VTE attenuated over time. In a crossover-cohort analysis, the odds of MI (OR 1.68 [95% CI 1.62–1.73]) and VTE (OR 3.30 [95% 3.19–3.41]) were higher in the 30 days following discharge after respiratory infection than during the 30-day baseline period. Hospitalization for respiratory infection was associated with increased risks of thrombosis that were highest in the first 30-days after discharge and declined over time.

## Introduction

Respiratory infections are a leading cause of morbidity and mortality worldwide and account for nearly 4.3 million deaths each year^1,2^. The morbidity of acute respiratory infections has been recently highlighted by the ongoing global Coronavirus disease (COVID-19) pandemic, which is associated with a substantial risk of thrombosis^3,4^. Infection with non-COVID-19 viral or bacterial respiratory pathogens is also associated with an excess risk of cardiovascular events^5–10^. Several studies have shown a higher risk of thrombotic events after respiratory illnesses, including bacterial (e.g. *Streptococcus pneumoniae)* and viral infections (e.g. influenza)^9–11^. However, prior reports are limited in size and duration of follow-up. Prior studies have not reported data on both arterial and venous events from the same cohort, nor have they evaluated multiple comparator groups (respiratory non-infectious, other infectious disease, or self-comparison prior to the event) to provide appropriate context for event rates observed after respiratory infection. Consequently, there is uncertainty regarding the magnitude and duration of these associations, particularly with respect to the risk of re-hospitalization for venous thrombosis after respiratory infection. To address this uncertainty, we evaluated the risk of arterial and venous thrombotic events after hospitalization for respiratory infections included in a large nationwide cohort of patients hospitalized in the United States in prior years. We assessed each patient's likelihood of hospitalization for a thrombotic event after discharge for respiratory infection as compared to a time period prior to the infection, and compared rates of readmissions between patients admitted with a non-infectious respiratory disease (asthma) and those admitted with a non-respiratory infectious disease (cellulitis).

## Methods

### Study population

Adults ≥ 18 years of age admitted to hospitals between 2012 and 2014 with a diagnosis of a respiratory infection were identified from the United States (US) Agency for Healthcare Research and Quality (AHRQ) Healthcare Cost and Utilization Project’s (HCUP) Nationwide Readmission Database (NRD). The NRD is a national administrative database of hospital discharge-level data; the 2014 NRD dataset includes 22 states and represents 51.2% of the US population and 49.3% of all US hospitalizations^12^. Respiratory infections were identified based on a primary or non-primary *International Classification of Diseases, Ninth Revision Clinical Modification* (ICD-9) diagnosis code for bacterial or viral pneumonia (Supplemental Table 1), based on prior definitions^13–15^. Patients hospitalized with non-infectious respiratory disease (asthma) or a non-respiratory skin and soft tissue infection (cellulitis) were selected as comparator (control) groups and identified by ICD-9 diagnosis codes in any position (Supplemental Table 1)^16,17^. For patients with multiple admissions during a calendar year, only the first hospitalization that met inclusion criteria was included in the final analysis. Patients discharged in the month of December during each year were excluded from the 30-day endpoint analysis due to incomplete follow-up data from the calendar-year files as per AHRQ guidelines^18^. Outcomes of 60, 90, 120 and 180-day readmission were evaluated in subsets of the overall cohort selected based on the month of discharge to permit the minimum follow up within each calendar year. Patients who died during the index hospitalization were excluded from all analyses. Demographics and comorbidities during the index hospitalization were defined by relevant ICD-9 diagnosis codes and standard AHRQ Elixhauser comorbidities.

### Outcomes

Thrombotic outcomes were evaluated in patients who survived to discharge after the index hospitalization. Hospital readmissions with acute myocardial infarction (AMI) and venous thromboembolism (VTE) were identified. Acute myocardial infarctions were identified using ICD-9 diagnosis codes for acute ST-segment elevation myocardial infarction (STEMI) (ICD-9 diagnosis codes 410.01 to 410.61, 410.81, and 410.91) and non-ST-segment elevation myocardial infarction (NSTEMI) (ICD-9 diagnosis code 410.71) in any position. Hospitalizations for venous thromboembolism (VTE) were defined by ICD-9 diagnosis codes for acute PE (415.1x) and lower extremity DVT (451.1x, 451.81, 453.2, and 453.4x) in any position. Emergency department visits that did not result in hospital admission were not included in this analysis.

The primary study outcome was 30-day hospital readmission with AMI or VTE, determined based on methodology described by HCUP^18^. Among patients with multiple MI or VTE readmissions within 30-days of the index hospital discharge, only the first relevant readmission was included for analysis.

### Statistical analysis

Continuous variables are reported as means with the standard error of measurement (SEM). Categorical variables are reported as percentages. Time-to-event Cox proportional hazards analyses were used to examine the association between respiratory infection and first re-hospitalization for MI or VTE, adjusted for clinical covariates including age, sex, tobacco use, hypertension, dyslipidemia, diabetes mellitus, prior coronary artery bypass grafting, prior percutaneous coronary intervention, congestive heart failure, peripheral vascular disorders, pulmonary circulation disorders, valvular heart disease, acquired immune deficiency syndrome, alcohol abuse, chronic blood loss anemia, chronic pulmonary disease, coagulopathy, deficiency anemias, depression, drug abuse, fluid and electrolyte disorders, hypothyroidism, liver disease, lymphoma, metastatic cancer, neurological disorders, obesity, paralysis, peptic ulcer disease, psychoses, renal failure, rheumatoid arthritis/collagen vascular disease, solid tumor without metastasis, weight loss, need for mechanical ventilation during hospitalization, primary insurance payer, and index hospital length of stay.

To characterize the increased thrombotic risk associated with hospitalization for respiratory infection, we performed a crossover-cohort analysis. This study design permits each patient to serve as his or her own control and reduces unmeasured confounding. We used conditional logistic regression to calculate the odds of MI or VTE during a 30-day follow-up period after the index hospitalization for respiratory infection compared to the risk of the event in the same patients during a 30-day period ending 1 week prior to the index hospital admission. Data from 1 week prior to the index admission were excluded from the baseline period in the crossover-cohort analysis due to the potential for effects of contamination from undiagnosed respiratory infections^19^. A case crossover analysis was also performed to estimate the odds of MI or VTE during a 90-day follow-up period after the index hospitalization for respiratory infection compared to the risk of the event in the same patients during a 90-day period ending 1 week prior to the index admission. Unweighted counts were used for all crossover-cohort analyses.

Sampling weights were applied to determine national incidence estimates unless otherwise specified^20^. Statistical analyses were performed using SPSS 26 (IBM SPSS Statistics, Armonk, NY). Statistical tests were two-sided and *P* values < 0.05 were considered to be statistically significant. The NRD is a publicly available, de-identified dataset, and consequently, the study did not require institutional board review. It was not possible to involve patients in the design, or conduct, or reporting, or dissemination plans of our research.

## Results

### Study population

We identified 2,248,366 records of patients who survived to discharge after a hospitalization with an ICD-9 diagnosis code for a respiratory infection during the study period. After applying sampling weights, this represents 5,271,068 patients nationally. Among the 898,450 patients (17.0%) with a respiratory infection pathogen specified, 626,924 had a bacterial respiratory infection and 271,525 had a viral respiratory pathogen. During this same 33-month time period, there were 1,765,101 unique patients without a respiratory infection who were hospitalized with a diagnosis of asthma and 1,258,549 hospitalized with cellulitis, corresponding to 4,067,290 and 2,902,620 patients nationwide, respectively, after applying sampling weights.

Baseline characteristics of patients hospitalized with respiratory infection, asthma, or cellulitis are shown in Table 1. Patients with respiratory infections were older, more likely to have heart failure, more likely to be current or former smokers, and less likely to be obese than patients hospitalized for asthma or cellulitis. Patients hospitalized with asthma were more likely to be female and to have chronic pulmonary disease, while diabetes mellitus with complications and peripheral vascular disorders were more prevalent in patients hospitalized with cellulitis (Table 1).

**Table 1:** Baseline characteristics of patients hospitalized with respiratory infection, asthma, and cellulitis.

	Respiratory infection	Asthma (without respiratory or skin infection)	Cellulitis (± asthma, without respiratory infection)
	(n = 5,271,068)	(n = 4,067,290)	(n = 2,902,620)
**Demographics**			
Age, years (S.E.)	67.3 (0.09)	53.5 (0.12)	58.0 (0.09)
Female sex	2,741,962 (52%)	2,921,719 (71.8%)	1,329,876 (45.8%)
**Cardiovascular risk factors and comorbidities**			
Tobacco use (current or former)	1,761,108 (33.4%)	1,244,734 (30.6%)	883,471 (30.4%)
Hypertension	3,184,633 (60.4%)	2,063,260 (50.7%)	1,601,206 (55.2%)
Dyslipidemia	1,737,877 (33%)	1,225,217 (30.1%)	839,399 (28.9%)
Diabetes mellitus, any	1,551,313 (29.4%)	963,316 (23.7%)	980,318 (33.8%)
Diabetes mellitus with chronic complications	309,094 (5.9%)	155,776 (3.8%)	365,088 (12.6%)
Diabetes mellitus, uncomplicated	1,251,397 (23.7%)	810,912 (19.9%)	620,941 (21.4%)
Prior coronary artery bypass grafting	307,309 (5.8%)	104,611 (2.6%)	134,057 (4.6%)
Prior percutaneous coronary intervention	271,513 (5.2%)	155,557 (3.8%)	107,050 (3.7%)
Congestive heart failure	1,053,613 (20%)	255,272 (6.3%)	308,567 (10.6%)
Peripheral vascular disorders	409,968 (7.8%)	157,999 (3.9%)	289,408 (10%)
Pulmonary circulation disorders	288,773 (5.5%)	66,581 (1.6%)	72,412 (2.5%)
Valvular heart disease	341,872 (6.5%)	130,034 (3.2%)	108,394 (3.7%)
**Other comorbidities**			
Acquired immune deficiency syndrome	7260 (0.1%)	8061 (0.2%)	8540 (0.3%)
Alcohol abuse	230,243 (4.4%)	178,953 (4.4%)	135,749 (4.7%)
Chronic blood loss anemia	56,539 (1.1%)	105,549 (2.6%)	25,591 (0.9%)
Chronic pulmonary disease	1,944,256 (36.9%)	3,607,628 (88.7%)	484,300 (16.7%)
Coagulopathy	408,088 (7.7%)	129,511 (3.2%)	148,214 (5.1%)
Deficiency anemias	1,371,042 (26%)	557,724 (13.7%)	596,613 (20.6%)
Depression	677,030 (12.8%)	637,739 (15.7%)	341,946 (11.8%)
Drug abuse	187,594 (3.6%)	255,151 (6.3%)	208,031 (7.2%)
Fluid and electrolyte disorders	2,225,362 (42.2%)	768,085 (18.9%)	774,357 (26.7%)
Hypothyroidism	757,625 (14.4%)	523,498 (12.9%)	326,663 (11.3%)
Liver disease	175,102 (3.3%)	122,187 (3%)	119,385 (4.1%)
Lymphoma	84,034 (1.6%)	18,330 (0.5%)	22,943 (0.8%)
Metastatic cancer	175,651 (3.3%)	45,457 (1.1%)	45,886 (1.6%)
Neurological disorders	619,899 (11.8%)	286,111 (7%)	214,192 (7.4%)
Obesity	670,815 (12.7%)	859,250 (21.1%)	631,276 (21.7%)
Paralysis	186,187 (3.5%)	63,466 (1.6%)	76,912 (2.6%)
Peptic ulcer disease	1912 (0%)	1286 (0%)	721 (0%)
Psychoses	300,242 (5.7%)	274,233 (6.7%)	163,891 (5.6%)
Renal failure	995,091 (18.9%)	316,193 (7.8%)	453,740 (15.6%)
Rheumatoid arthritis/collagen vascular disease	206,380 (3.9%)	146,904 (3.6%)	99,145 (3.4%)
Solid tumor without metastasis	175,288 (3.3%)	47,371 (1.2%)	53,005 (1.8%)
Weight loss	522,183 (9.9%)	98,115 (2.4%)	149,545 (5.2%)
**Hospital characteristics**			
*Hospital size*			
Small	865,210 (16.4%)	563,413 (13.9%)	427,540 (14.7%)
Medium	1,343,133 (25.5%)	1,021,981 (25.1%)	738,361 (25.4%)
Large	3,062,726 (58.1%)	2,481,895 (61%)	1,736,719 (59.8%)
*Hospital location / type*			
Rural	1,920,798 (36.4%)	1,320,842 (32.5%)	1,061,360 (36.6%)
Urban non-teaching	2,461,805 (46.7%)	2,405,587 (59.1%)	1,469,776 (50.6%)
Urban teaching	888,465 (16.9%)	340,861 (8.4%)	371,484 (12.8%)
Payer type*			
Medicare	3,513,109 (66.8%)	1,653,323 (40.7%)	1,376,232 (47.5%)
Medicaid	538,043 (10.2%)	836,140 (20.6%)	434,989 (15%)
Private insurance	849,115 (16.1%)	1,198,472 (29.5%)	679,119 (23.4%)
Self-pay	199,227 (3.8%)	197,487 (4.9%)	247,899 (8.6%)
No charge	24,780 (0.5%)	29,074 (0.7%)	33,879 (1.2%)
Other	137,324 (2.6%)	145,614 (3.6%)	124,617 (4.3%)

All *P* values < 0.001.

*Data available for a subset of 99.8% of cases (n = 12,218,443).

### 30-day hospital readmission

**Figure 1:**
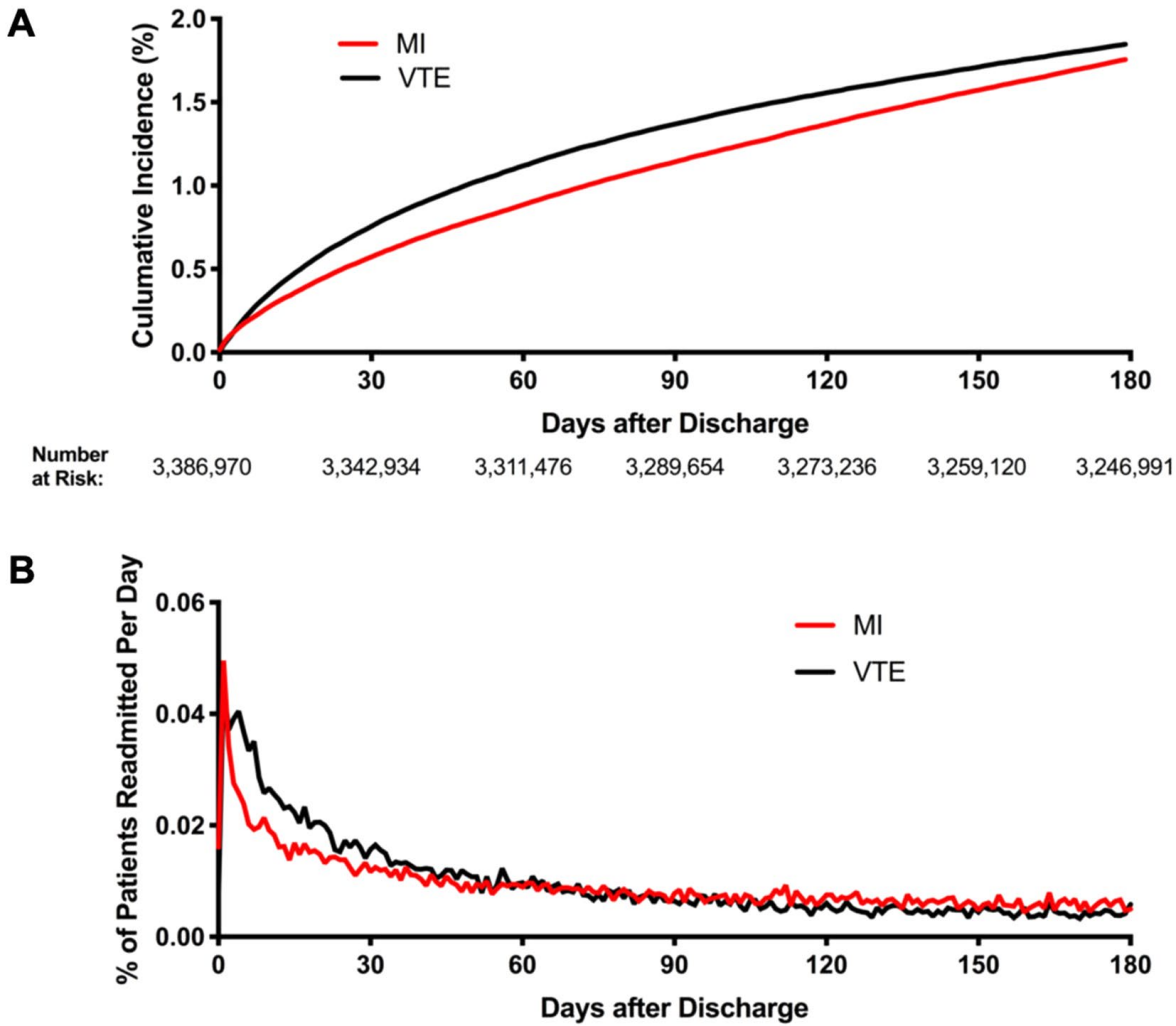
Cumulative incidence of first readmission for MI and VTE in the first 180 days after discharge following respiratory infection (Panel **A**) and the daily proportion of patients with readmission for MI and VTE (Panel **B**).

Hospital readmissions for MI and VTE were greatest early after discharge for respiratory infection. Among patients hospitalized with respiratory infections who were discharged from their index admission, 0.56% of patients were readmitted with MI and 0.78% were readmitted with VTE within 30 days. The cumulative incidence of first readmission for MI and VTE in the first 180 days after discharge following respiratory infection are shown in Fig. 1A**.** By 180 days following discharge after respiratory infection, 1.49% of patients were readmitted with MI and 1.65% were readmitted with VTE. The daily proportion of patients with readmission for MI and VTE after respiratory infection are shown in Fig. 1B. Cumulative rates of first re-hospitalization with MI and VTE following respiratory infection are shown at multiple time points in Supplemental Table 2.

**Figure 2:**
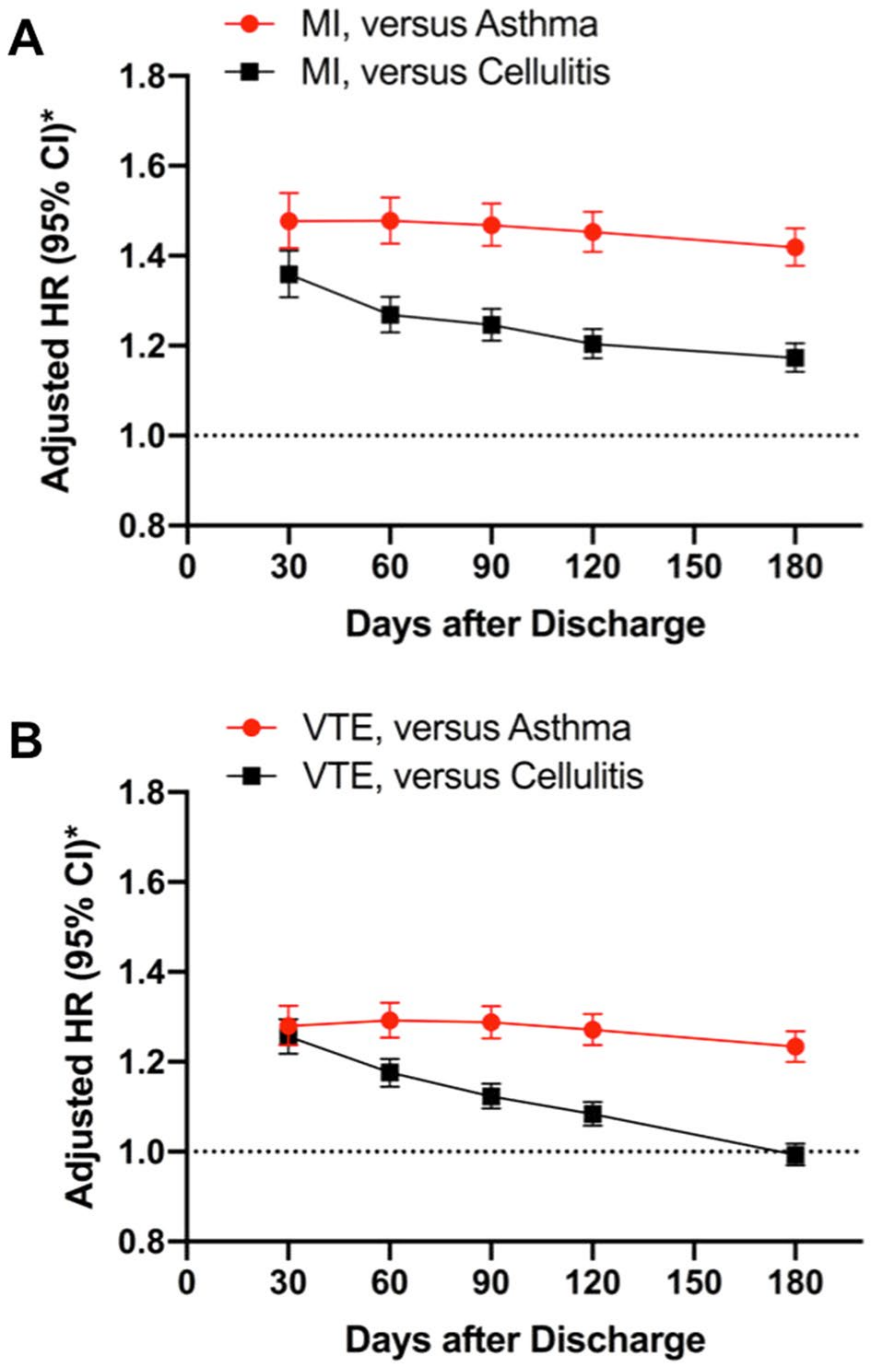
Adjusted hazard of readmission for myocardial infarction [MI] (Panel **A**) and venous thromboembolism [VTE] (Panel **B**) over time after hospital discharge among patients with respiratory infection versus those with asthma (red lines) and cellulitis (black lines). *Cox proportional hazards analyses were adjusted for clinical covariates including age, sex, tobacco use, hypertension, dyslipidemia, diabetes mellitus, prior coronary bypass grafting, prior percutaneous coronary intervention, congestive heart failure, peripheral vascular disorders, pulmonary circulation disorders, valvular heart disease, acquired immune deficiency syndrome, alcohol abuse, chronic blood loss anemia, chronic pulmonary disease, coagulopathy, deficiency anemia, depression, drug abuse, fluid and electrolyte disorders, hypothyroidism, liver disease, lymphoma, metastatic cancer, neurological disorders, obesity, paralysis, peptic ulcer disease, psychoses, renal failure, rheumatoid arthritis/collagen vascular disease, solid tumor without metastasis, weight loss, need for mechanical ventilation during hospitalization, primary insurance payer, and index hospital length of stay.

Patients with respiratory infection were more likely to be readmitted for MI or VTE compared to patients initially admitted with asthma or cellulitis. Relative to patients hospitalized for a comparator condition of asthma or cellulitis, patients with respiratory infection had an adjusted hazard of readmission for MI (adjusted HR [aHR] 1.48, 95% CI 1.42–1.54 vs. asthma; aHR 1.36, 95% CI 1.31–1.41 vs. cellulitis). Similarly, patients with respiratory infection had an adjusted hazard of readmission for VTE relative to comparator conditions (aHR 1.28, 95% CI 1.24–1.33 vs. asthma; aHR 1.26, 95% CI 1.22–1.30 vs. cellulitis). The hazard of readmission for MI after hospitalization for respiratory infection was greater than for patients hospitalized with asthma or cellulitis at all time points evaluated (Fig. 2A); the hazard of readmission for VTE after respiratory infection was greater at time points before 180 days (Fig. 2B). In all analyses, the incremental hazard for readmission for thrombotic events associated with respiratory infection was greatest in the first 30 days after discharge and was attenuated at 180-day follow-up.

### Crossover-cohort analysis

**Figure 3:**
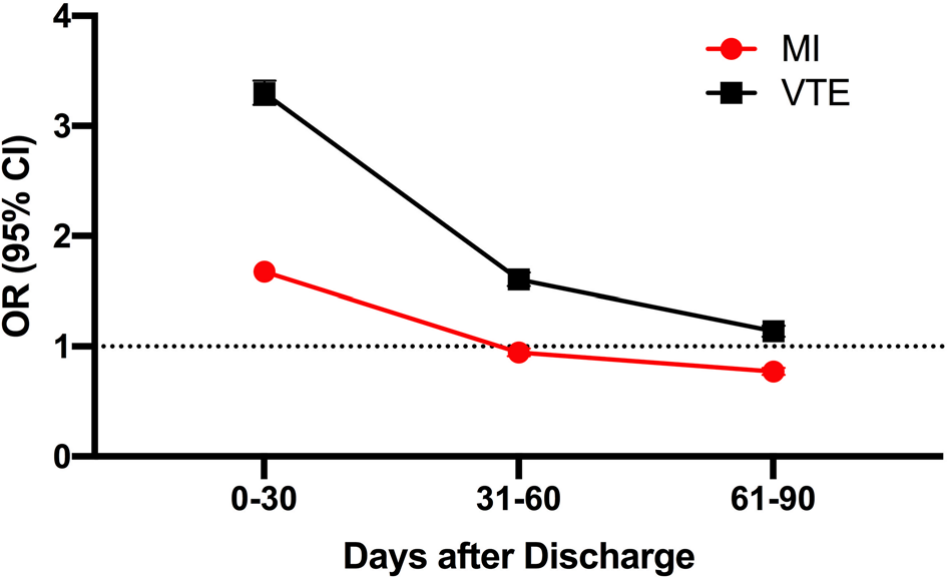
Crossover-cohort analysis comparing the odds of hospitalization for a thrombotic event by time period after respiratory infection versus a 30-day baseline period prior to infection.

In the crossover-cohort analysis, where each patient serves as his or her own control, there were significantly more thrombotic events after hospitalization for a respiratory infection than during a 30-day period prior to the infection. For the outcome of MI, there were 57 events per 10,000 discharges after a respiratory infection versus 34 events per 10,000 discharges prior to a respiratory infection, corresponding to an absolute increase risk difference of 23 (95% CI 21 to 24) per 10,000 discharges and an odds ratio of 1.68 (95% CI 1.62–1.73). For the outcome of VTE, there were 79 events per 10,000 discharges after a respiratory infection versus 24 events per 10,000 discharges prior to a respiratory infection, corresponding to an absolute increase risk difference of 55 (95% CI 54 to 56) per 10,000 discharges and an odds ratio of 3.30 (95% CI 3.19 to 3.41 (Table 2). Similar findings were observed in a crossover-cohort analysis when a 90-day period following discharge was compared to the 90-day baseline period ending 1 week prior to the index admission for respiratory infection (Supplemental Table 3). The incremental risk of thrombosis associated with hospitalization for respiratory infection was highest at 30-days and attenuated over time for both MI and VTE (Fig. 3, Supplemental Fig. 2).

**Table 2:** Crossover-cohort analysis comparing the risk of thrombotic events in the 30-day period after discharge following respiratory infection versus the 30-day period ending 7 days prior to admission for respiratory infection.

	30-day period after discharge following respiratory infection	30-day period ending 7 days prior to admission for respiratory infection	Absolute difference (%, 95% CI)	Odds ratio* (95% CI)
Any respiratory infection (1,895,121)†				
Any MI	0.57%	0.34%	0.23% (0.21–0.24%)	1.68 (1.62–1.73)
Any VTE	0.79%	0.24%	0.55% (0.54–0.56%)	3.30 (3.19–3.41)
Bacterial pathogen specified (n = 225,385)†				
Any MI	0.51%	0.35%	0.16% (0.12–0.20%)	1.46 (1.33–1.60)
Any VTE	0.90%	0.22%	0.68% (0.64–0.72%)	4.08 (3.70–4.50)
Viral pathogen specified (n = 71,259)†				
Any MI	0.37%	0.15%	0.22% (0.17–0.27%)	2.44 (1.95–3.05)
Any VTE	0.48%	0.11%	0.37% (0.32–0.43%)	4.49 (3.50–5.76)

^†^Unweighted counts and proportions. *Conditional logistic regression of unweighted data for case-cohort analysis.

Among patients with a respiratory pathogen identified, there were 37 MI per 10,000 discharges after a viral respiratory infection versus 15 per 10,000 prior to the infection, corresponding to an absolute increase risk difference of 22 (95% CI 17 to 27) MI per 10,000 and an odds ratio of 2.44 (95% CI 1.95–3.05) (Table 2). For the outcome of VTE, there were 48 events per 10,000 discharges after a viral respiratory infection versus 11 events per 10,000 prior to the infection, corresponding to an absolute increase risk difference of 37 (95% CI 32 to 43) events per 10,000 and an odds ratio of 4.49 (95% CI 3.50–5.76). Thrombotic risks after discharge in patients with respiratory infection due to bacterial pathogens were also elevated and are shown in Table 2. As in the overall cohort of patients, the odds of a thrombotic event following hospitalization for viral and bacterial respiratory infections were greatest in the first 30-days after discharge and attenuated over time (Supplemental Fig. 1).

## Discussion

In this analysis of patients hospitalized with a respiratory infection nationwide, 30-day readmission for MI and VTE occurred in 56 per 10,000 discharges and 78 per 10,000 discharges, respectively. By 180 days, readmission for MI occurred in 149 per 10,000 discharges and readmission for VTE occurred in 165 per 10,000 discharges. Relative to non-infectious (asthma) and non-respiratory infectious (cellulitis) comparator conditions, respiratory infection was associated with a greater age and sex-adjusted hazard of 30-day readmission for MI and VTE. In a crossover-cohort analysis, the odds of MI and VTE were higher in the first 30 days following hospital discharge after respiratory infection than during a similar pre-admission period (Summary Figure). Among patients with a pathogen identified, the increased odds of MI and VTE after compared to before the respiratory infection were particularly pronounced for viral respiratory illness.

The exact mechanisms of thrombotic risk in patients with a recent infection are not fully understood. Our comparative analyses make clear that immobility is not the only concern. Inflammation may play a role in cardiovascular events after respiratory infection, and elevated IL-6 and IL-10 cytokine concentrations at hospital discharge have been associated with early cardiovascular mortality^21^. Platelet activation in the setting of infection may also mediate thrombotic events. In a study of 278 adults with community-acquired pneumonia, elevations in plasma soluble CD40 ligand and soluble P-selectin concentrations were independent predictors of subsequent MI^22^. In patients hospitalized with COVID-19, the risk of thrombosis is markedly elevated^4,23^, and autopsies demonstrate extensive platelet–fibrin thrombi in multiple organs with COVID-19. However, the pathogenesis of the pro-thrombotic state associated with bacterial and viral infections is not fully understood.

The findings of the present analysis are consistent with prior reports suggesting an increased risk of thrombotic events associated with respiratory infection^6,7,9,11,24^. In a study of 1,271 patients with and without pneumonia identified from community-based cohorts, there was an increased hazard of cardiovascular events early after respiratory illness that persisted beyond 1 year, but that study was far smaller in size and did not address VTE or MI specifically^7^. To our knowledge, this is the largest contemporary series to evaluate MI risk after hospitalization after respiratory infection, and the first to place thrombotic risks in context with other common indications for hospital admission.

Our results demonstrating an increased risk of VTE after respiratory infection also provide new insights into the magnitude of thrombotic risks in this cohort, as data on risks of VTE after respiratory infection are limited^25–27^. In a prior matched case-control study of 11,557 patients treated for VTE, the excess risk of a deep vein thrombosis associated with a respiratory infection persisted to 1 year, with the greatest risk in the first 30 days after infection^26^. However, crossover-cohort study designs evaluating the risks of incident VTE overall and by subtype of respiratory infection have not previously been reported. We demonstrate that the increased risk of VTE is related to respiratory infection, and not to simply due to comorbid characteristics. Furthermore, our analyses with hospitalizations for comparator conditions (asthma or cellulitis) demonstrate that thrombotic risks associated with respiratory infection cannot be attributed to hospitalization in general. Although extended-duration thromboprophylaxis after hospital discharge for acute medical illness is associated with lower risk of VTE in multiple randomized trials, optimal management strategies to mitigate thrombotic risks after infection have not yet been defined^28,29^.

This study has several strengths, including its large size, cohort study design, and use of validated criteria for the incidence of acute MI and VTE. It is the first to evaluate both arterial and venous thrombosis after hospitalization for respiratory illness and follow patients out to 6 months from a large, representative cohort from the United States. Additionally, this study uses both respiratory non-infectious and non-respiratory infectious comparator admissions, and evaluates thrombotic risks associated with both viral and bacterial respiratory infections from the same cohort.

There are a number of important limitations to the current data. Data from the NRD are retrospectively derived from ICD-9 diagnosis and procedure codes recorded in state administrative databases and may be subject to reporting bias and coding errors. Still, the NRD provides data on a large, representative, national cohort of hospitalized patients with different forms of the disease. We used a validated definition of respiratory infection to identify index hospitalizations, but discrete clinical data, such as results of clinical imaging and microbiology laboratory data were not captured in this administrative dataset. We cannot exclude residual confounding in adjusted models assessing risks of thrombosis associated with respiratory illness relative to comparator conditions. However, the crossover-cohort analysis mitigates this concern, since each patient serves as his or her own control. In-hospital and discharge medications were not recorded, and associations between antithrombotic therapy, anti-infective therapy, and readmission with MI and VTE could not be determined. Relatively few patients (17%) had ICD-9 coding specifying the pathogen causing respiratory infection, limiting the size of viral and bacterial subgroups for analysis. Patients who had a diagnosis code for a respiratory infection and asthma or a cellulitis during the same hospital admission were included in the analysis as a respiratory infection and were not included in the corresponding comparator groups. We cannot be certain that some asthma hospitalizations were not due to unrecognized respiratory infections. Nevertheless, patients with non-infectious respiratory disease (asthma) and infectious non-respiratory disease (cellulitis) are meaningful comparators to assess thrombotic risk associated with respiratory infection. Thrombotic events that did not require hospital admission and deaths that occurred out-of-hospital could not be captured in this database of inpatient hospitalizations. Readmission data were limited to 180 days; longer-term rates of hospital readmission could not be determined because patients in NRD are not linked across calendar years. Finally, years 2012–2014 were selected as they are the most recent full calendar years in which ICD-9 diagnosis codes were used.

## Conclusion

In conclusion, hospitalization for respiratory infection was associated with an increased risk of arterial and venous thrombosis that was highest in the first 30-days after discharge and declined over time. Among patients with a pathogen identified, the odds of MI and VTE at 30 days were especially high in patients with recent viral respiratory illness. Strategies to mitigate thrombotic risk in patients discharged following respiratory infection should be tested in clinical trials.

## Supplementary Information

Supplementary Information

## Data Availability

The NRD is a publicly available, de-identified dataset available from the AHRQ Healthcare Cost and Utilization Project.

## Abbreviations

AHRQ: Agency for Healthcare Research and Quality
HCUP: Healthcare Cost and Utilization Project’s
ICD: International Classification of Disease
MI: Myocardial Infarction
NRD: Nationwide Readmission Database
VTE: Venous Thromboembolism
