# Supplementary material for: Risk of thrombotic events after respiratory infection requiring hospitalization
Source: Sci Rep. 2021 Feb 18;11:4053. doi: 10.1038/s41598-021-83466-9 (PMC7893015; doi:10.1038/s41598-021-83466-9)
Supplement: Supplementary file 1 — Supplementary Information [file 41598_2021_83466_MOESM1_ESM.docx]

**Risk of Thrombotic Events after Respiratory Infection Requiring Hospitalization**

Nathaniel R. Smilowitz MD, MS ^1,2^, Varun Subashchandran BS ^1^, Jonathan Newman MD, MPH^1^, Michael E. Barfield, MD ^3^, Thomas S. Maldonado MD ^3^, Shari B. Brosnahan, MD ^4^, Eugene Yuriditsky MD ^1^, James M. Horowitz MD ^1^, Binita Shah MD, MS ^1,2^, Harmony R. Reynolds MD ^1^, Judith S. Hochman MD ^1^, Jeffrey S. Berger MD, MS ^1,3^

*^1^ Leon H. Charney Division of Cardiology, Department of Medicine, New York University School of Medicine, New York, NY*

*^2^ Department of Medicine, VA New York Harbor Healthcare System, New York, NY*

*^3^ Department of Surgery, New York University School of Medicine, New York, NY*

*^4^ NYU Division of Pulmonary, Critical Care, and Sleep Medicine, Department of Medicine, New York University School of Medicine, New York, NY*

**SUPPLEMENTAL MATERIALS**

**Supplemental Table 1.**

| **Group** | **ICD-9 Code** | **Description** |
| --- | --- | --- |
| **Respiratory Infection** | 0796 | Respiratory syncytial virus (RSV) |
|  | 4800 | Pneumonia due to adenovirus |
|  | 4801 | Pneumonia due to respiratory syncytial virus |
|  | 4802 | Pneumonia due to parainfluenza virus |
|  | 4803 | Pneumonia due to SARS-associated coronavirus |
|  | 4808 | Pneumonia due to other virus not elsewhere classified |
|  | 4809 | Viral pneumonia, unspecified |
|  | 481 | Pneumococcal pneumonia [Streptococcus pneumoniae pneumonia] |
|  | 4820 | Pneumonia due to Klebsiella pneumoniae |
|  | 4821 | Pneumonia due to Pseudomonas |
|  | 4822 | Pneumonia due to Hemophilus influenzae [H. influenzae] |
|  | 48230 | Pneumonia due to Streptococcus, unspecified |
|  | 48231 | Pneumonia due to Streptococcus, group A |
|  | 48232 | Pneumonia due to Streptococcus, group B |
|  | 48239 | Pneumonia due to other Streptococcus |
|  | 48240 | Pneumonia due to Staphylococcus, unspecified |
|  | 48241 | Methicillin susceptible pneumonia due to Staphylococcus aureus |
|  | 48242 | Methicillin resistant pneumonia due to Staphylococcus aureus |
|  | 48249 | Other Staphylococcus pneumonia |
|  | 48281 | Pneumonia due to anaerobes |
|  | 48282 | Pneumonia due to Escherichia coli [E. coli] |
|  | 48283 | Pneumonia due to other gram-negative bacteria |
|  | 48284 | Pneumonia due to Legionnaires' disease |
|  | 48289 | Pneumonia due to other specified bacteria |
|  | 4829 | Bacterial pneumonia, unspecified |
|  | 4830 | Pneumonia due to mycoplasma pneumoniae |
|  | 4831 | Pneumonia due to chlamydia |
|  | 4838 | Pneumonia due to other specified organism |
|  | 4841 | Pneumonia in cytomegalic inclusion disease |
|  | 4843 | Pneumonia in whooping cough |
|  | 4845 | Pneumonia in anthrax |
|  | 4846 | Pneumonia in aspergillosis |
|  | 4847 | Pneumonia in other systemic mycoses |
|  | 4848 | Pneumonia in other infectious diseases classified elsewhere |
|  | 485 | Bronchopneumonia, organism unspecified |
|  | 486 | Pneumonia, organism unspecified |
|  | 4870 | Influenza with pneumonia |
|  | 4871 | Influenza with other respiratory manifestations |
|  | 4878 | Influenza with other manifestations |
|  | 48801 | Influenza due to identified avian influenza virus with pneumonia |
|  | 48802 | Influenza due to identified avian influenza virus with other respiratory manifestations |
|  | 48809 | Influenza due to identified avian influenza virus with other manifestations |
|  | 48811 | Influenza due to identified 2009 H1N1 influenza virus with pneumonia |
|  | 48812 | Influenza due to identified 2009 H1N1 influenza virus with other respiratory manifestations |
|  | 48819 | Influenza due to identified 2009 H1N1 influenza virus with other manifestations |
|  | 48881 | Influenza due to identified novel influenza A virus with pneumonia |
|  | 48882 | Influenza due to identified novel influenza A virus with other respiratory manifestations |
|  | 48889 | Influenza due to identified novel influenza A virus with other manifestations |
|  | 46611 | Acute bronchiolitis due to respiratory syncytial virus (RSV) |
|  | 4658 | Acute upper respiratory infections of other multiple sites |
|  | 4659 | Acute upper respiratory infections of unspecified site |
| **Asthma** | 49300 | Extrinsic asthma, unspecified |
|  | 49301 | Extrinsic asthma with status asthmaticus |
|  | 49302 | Extrinsic asthma with (acute) exacerbation |
|  | 49310 | Intrinsic asthma, unspecified |
|  | 49311 | Intrinsic asthma with status asthmaticus |
|  | 49312 | Intrinsic asthma with (acute) exacerbation |
|  | 49320 | Chronic obstructive asthma, unspecified |
|  | 49321 | Chronic obstructive asthma with status asthmaticus |
|  | 49322 | Chronic obstructive asthma with (acute) exacerbation |
|  | 49381 | Exercise induced bronchospasm |
|  | 49382 | Cough variant asthma |
|  | 49390 | Asthma, unspecified type, unspecified |
|  | 49391 | Asthma, unspecified type, with status asthmaticus |
|  | 49392 | Asthma, unspecified type, with (acute) exacerbation |
| **Cellulitis** | 035 | Erysipelas |
|  | 68100 | Cellulitis and abscess of finger, unspecified |
|  | 68101 | Felon |
|  | 68102 | Onychia and paronychia of finger |
|  | 68110 | Cellulitis and abscess of toe, unspecified |
|  | 68111 | Onychia and paronychia of toe |
|  | 6819 | Cellulitis and abscess of unspecified digit |
|  | 6820 | Cellulitis and abscess of face |
|  | 6821 | Cellulitis and abscess of neck |
|  | 6822 | Cellulitis and abscess of trunk |
|  | 6823 | Cellulitis and abscess of upper arm and forearm |
|  | 6824 | Cellulitis and abscess of hang, except fingers and thumb |
|  | 6825 | Cellulitis and abscess of buttock |
|  | 6826 | Cellulitis and abscess of leg, except foot |
|  | 6827 | Cellulitis and abscess of foot, except toes |
|  | 6828 | Cellulitis and abscess of other specified sites |
|  | 6829 | Cellulitis and abscess of unspecified sites |
|  | 72886 | Necrotizing fasciitis |
|  | 6800 | Carbuncle and furuncle of face |
|  | 6801 | Carbuncle and furuncle of neck |
|  | 6802 | Carbuncle and furuncle of trunk |
|  | 6803 | Carbuncle and furuncle of upper arm and forearm |
|  | 6804 | Carbuncle and furuncle of hand |
|  | 6805 | Carbuncle and furuncle of buttock |
|  | 6806 | Carbuncle and furuncle of leg, except foot |
|  | 6807 | Carbuncle and furuncle of foot |
|  | 6808 | Carbuncle and furuncle of other specified sites |
|  | 6809 | Carbuncle and furuncle of unspecified site |
|  | *684* | Impetigo |
|  | 68600 | Pyoderma, unspecified |
|  | 68601 | Pyoderma gangrenosum |
|  | 68609 | Other pyoderma |
|  | 6861 | Pyogenic granuloma of skin and subcutaneous tissue |
|  | 6868 | Other specified local infections of skin and subcutaneous tissue |
|  | 6869 | Unspecified local infection of skin and subcutaneous tissue |
|  | 7048 | Other specified diseases of hair and hair follicles |

**Supplemental Table 2.** Absolute frequency of readmission with myocardial infarction and venous thromboembolism at 30, 60, 90, 120 and 180 days following discharge after a hospitalization with respiratory infection.

|  | **Readmission with Myocardial Infarction** | | |  | | **Readmission with Venous Thromboembolism** | | | |
| --- | --- | --- | --- | --- | --- | --- | --- | --- | --- |
|  | **Respiratory Infection** | **Asthma** | **Cellulitis** |  | **Respiratory Infection** | | **Asthma** | **Cellulitis** |  |
| **30-Day** | 0.56% | 0.22% | 0.31% |  | 0.78% | | 0.42% | 0.51% |  |
| **60-Day*** | 0.86% | 0.35% | 0.52% |  | 1.13% | | 0.62% | 0.79% |  |
| **90-Day*** | 1.11% | 0.46% | 0.69% |  | 1.36% | | 0.76% | 1.00% |  |
| **120-Day*** | 1.32% | 0.56% | 0.86% |  | 1.53% | | 0.88% | 1.18% |  |
| **180-Day*** | 1.69% | 0.76% | 1.18% |  | 1.78% | | 1.08% | 1.54% |  |

* Outcomes at 60, 90, 120 and 180-days were evaluated in subsets of the overall cohort based on the minimum follow up required. Weighted proportions are shown.

**Supplemental Table 3.** Crossover-cohort analysis comparing the risk of thrombotic events in the 90-day period after discharge following respiratory infection versus the 90-day period ending 7 days prior to admission for respiratory infection.

|  | **90-day period after discharge following respiratory infection** | **90-day period ending 7 days prior to admission for respiratory infection** | **Absolute Difference (%, 95% CI)** | **Odds Ratio* (95% CI)** |
| --- | --- | --- | --- | --- |
| **Any Respiratory Infection (1,046,798) †** | |  |  |  |
| **Any MI** | 1.11% | 0.73% | 0.37% (0.35-0.40%) | 1.52 (1.47– 1.56) |
| **Any VTE** | 1.40% | 0.50% | 0.90% (0.87-0.93%) | 2.82 (2.73 – 2.90) |

† Unweighted counts and proportions. Analysis restricted to patients discharged from March to September to ensure complete 90-day follow up. * Conditional logistic regression of unweighted data for case-cohort analysis.**Supplemental Figure 1:** Crossover-cohort analysis comparing the odds of hospitalization for a thrombotic event by time period after bacterial respiratory infection (**Panel A**) and viral respiratory infection (**Panel B**) versus a 30-day baseline period prior to infection.

**Panel A: Bacterial respiratory infection**

**
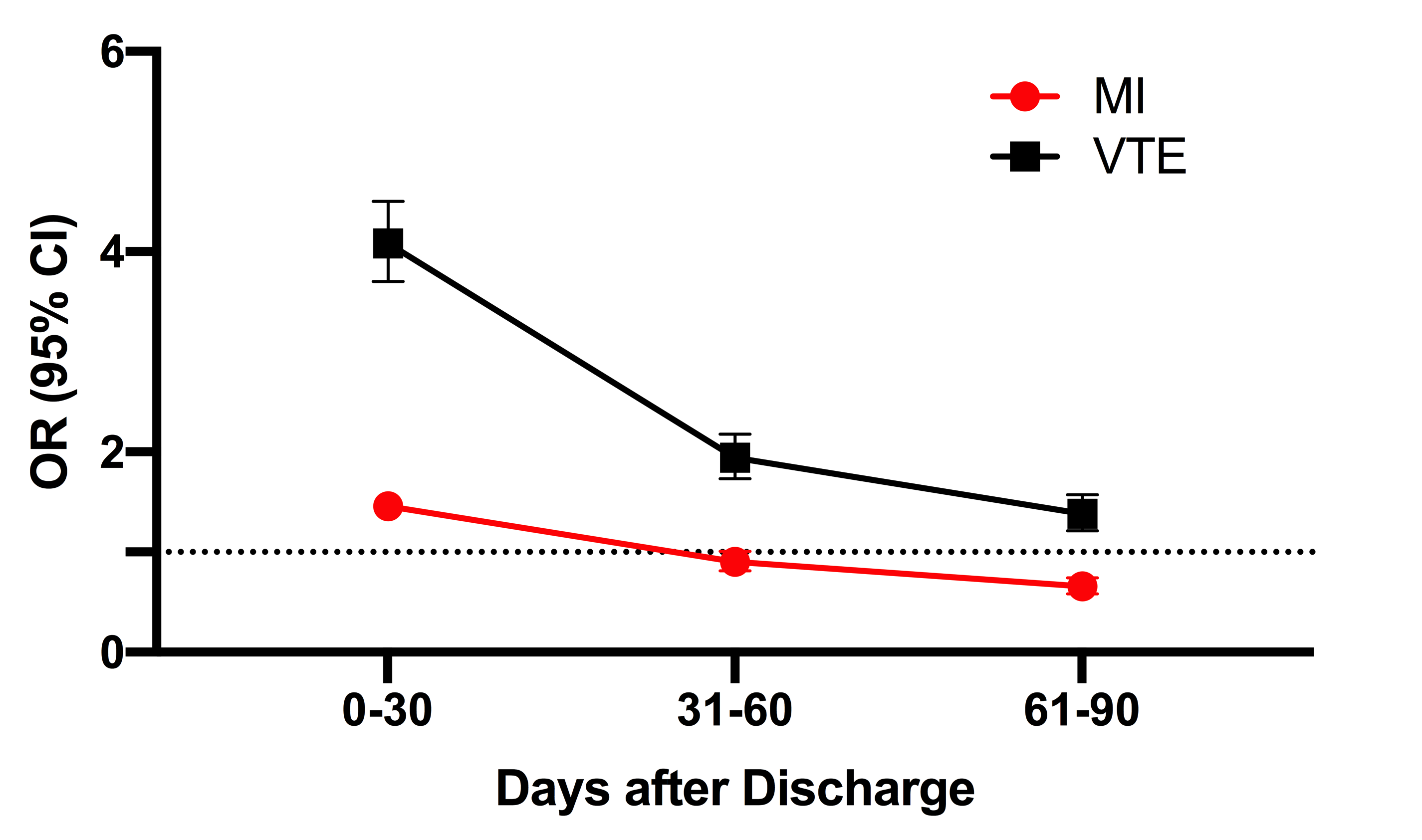
**

**Panel B: Viral respiratory infection
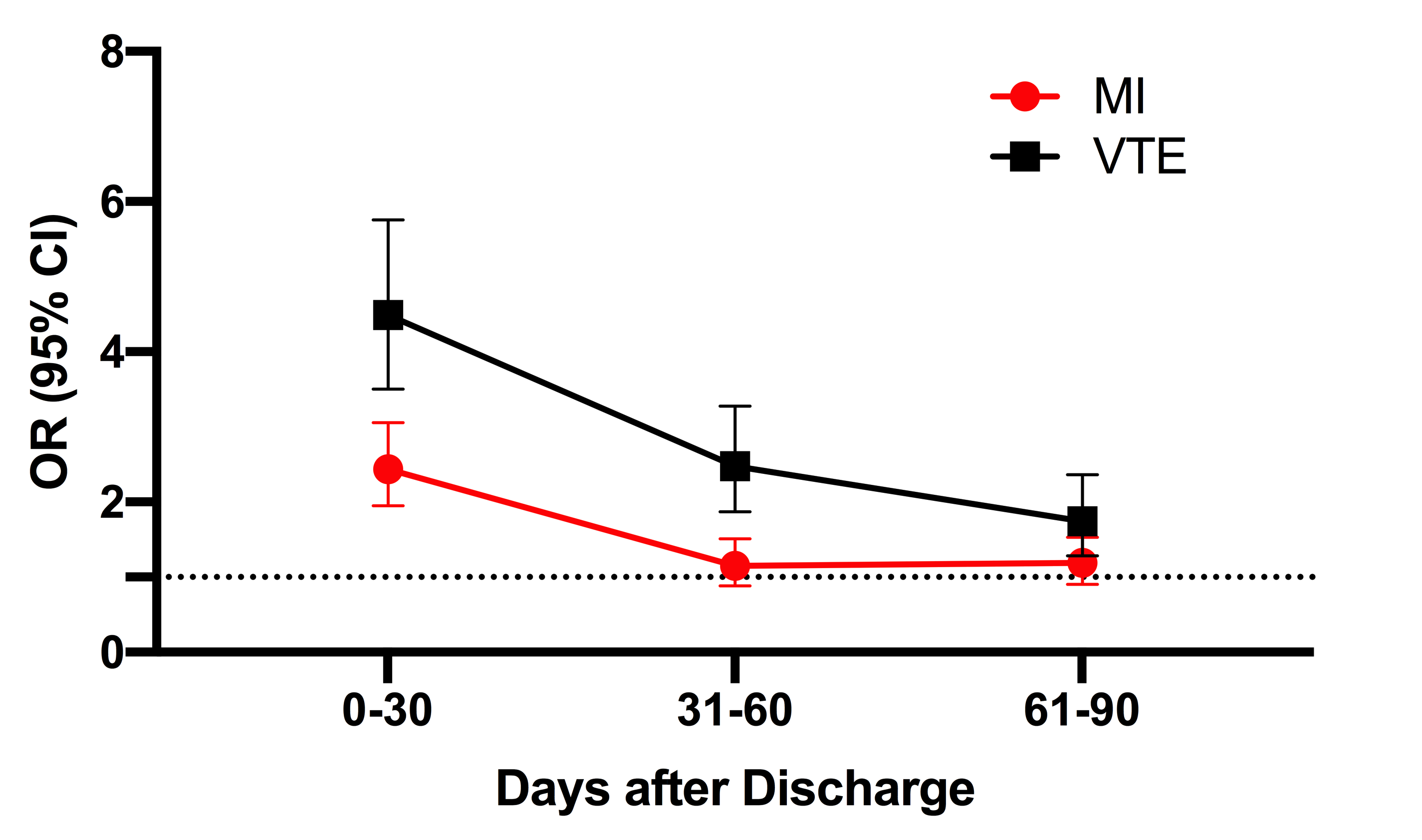
**

**Supplemental Figure 2:** Summary figure depicting the study design and key findings.**
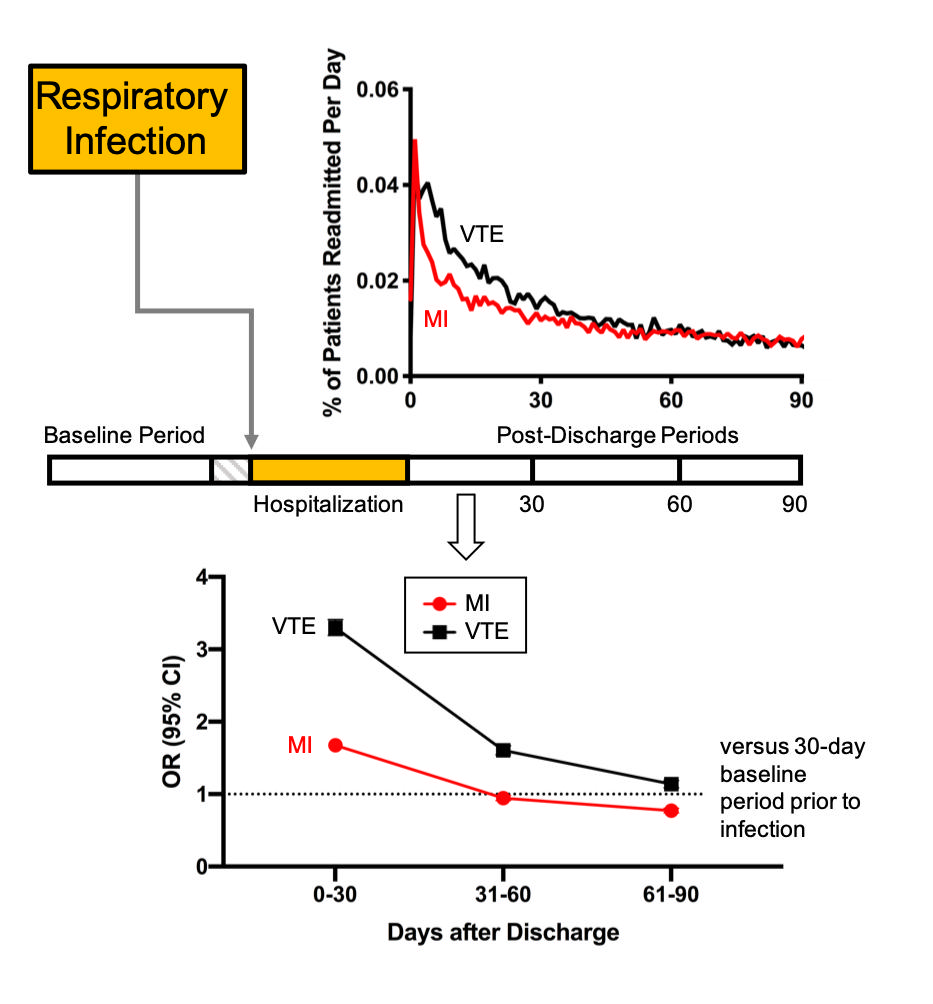
**
